# Rapid and reliable diagnosis of mucormycosis using colorimetric loop-mediated isothermal amplification

**DOI:** 10.1128/jcm.01790-25

**Published:** 2026-02-26

**Authors:** Yiyou Gu, Belal A. Ibrahim, Teclegiorgis Gebremariam, Sondus Alkhazraji, Robina Aerts, Yuri Vanbiervliet, Katrien Lagrou, Johan Maertens, Jürgen Prattes, Matthias Egger, Karl Dichtl, Jeffrey D. Jenks, Martin Hoenigl, Ashraf S. Ibrahim

**Affiliations:** 1 The Lundquist Institute at Harbor-University of California Los Angeles (UCLA) Medical Center 21640, Torrance, California, USA; 2 Vitalex Biosciences LLC 840912 , Trabuco Canyon, California, USA; 3 Department of Internal Medicine, University Hospitals Leuven 60182 , Leuven, Belgium; 4 Department of Microbiology Immunology and Transplantation, KU Leuven 573654 , Leuven, Belgium; 5 Department of Laboratory Medicine, National Reference Center for Mycosis, University Hospitals Leuven 60182 , Leuven, Belgium; 6 Department of Hematology, University Hospitals Leuven 60182 , Leuven, Belgium; 7 Division of Infectious Diseases, Department of Medicine, Translational Mycology Research Center, Excellence Centre for Medical Mycology (ECMM), Medical University of Graz 31475, Graz, Austria; 8 Diagnostic and Research Institute of Hygiene, Microbiology and Environmental Medicine, Medical University of Graz 31475, Graz, Austria; 9 Durham County Department of Public Health, Durham, North Carolina, USA; 10 Division of Infectious Diseases, Department of Medicine, Duke University 169103, Durham, North Carolina, USA; 11 David Geffen School of Medicine at UCLA 12222, Los Angeles, California, USA; University of Utah, Salt Lake City, Utah, USA

**Keywords:** mucormycosis, diagnosis, LAMP, BAL, *Rhizopus*

## Abstract

Current diagnostic approaches for mucormycosis are often limited by low sensitivity and prolonged turnaround times, which result in delayed treatment and poor clinical outcomes. We developed a novel diagnostic method utilizing a colorimetric loop-mediated isothermal amplification (LAMP) assay for the rapid and sensitive detection of mucormycosis. The assay incorporates specifically designed primers capable of detecting as low as 0.001 picograms (pg) of spiked genomic DNA from Mucorales fungi. This LAMP assay demonstrated a high sensitivity of 98% and a 100% specificity of detecting fungal ribosomal DNA (rDNA) in bronchoalveolar lavage (BAL) samples collected from mice infected with Mucorales fungi (*n* = 48) or from an uninfected control group (*n* = 15). To align the assay with clinical antifungal therapy, a subset of infected mice was treated with either liposomal amphotericin B (LAMB) or a combination of LAMB and a humanized monoclonal antibody (VX-01) targeting the Mucorales-specific surface protein CotH3. Consistent with the treatment efficacy, the LAMP assay detected significantly lower fungal burdens in BAL samples from mice receiving the combination therapy compared to those treated with LAMB alone or placebo. Further validation was conducted using BAL samples from patients diagnosed with mucormycosis (*n* = 24) or aspergillosis (*n* = 17). The assay demonstrated a sensitivity of 79% and a specificity of 94%. These findings highlight the diagnostic potential of this LAMP-based assay as a point-of-care. Its high sensitivity, specificity, and rapid turnaround time position this assay as a promising tool for early and accurate detection of mucormycosis, with the potential to improve patient management and clinical outcomes.

**IMPORTANCE:**

Mucormycosis is a rapidly progressive and fatal fungal infection. Timely diagnosis is critical for effective treatment, yet current diagnostic tools are slow, insensitive, or require complex laboratory procedures. In this study, we developed and validated a colorimetric loop-mediated isothermal amplification (LAMP) assay that enables rapid and reliable detection of Mucorales DNA directly from bronchoalveolar lavage (BAL) specimens. The assay demonstrated high sensitivity and specificity in both experimental mouse models and clinical samples, producing results within 1 h without the need for sophisticated equipment. This simple, robust, and cost-effective molecular diagnostic tool holds great potential for early detection of mucormycosis, facilitating prompt antifungal therapy and improving patient survival.

## INTRODUCTION

Mucormycosis is an aggressive and often fatal fungal infection caused by filamentous fungi of the order Mucorales (1). These opportunistic pathogens are commonly found in soil and decaying organic matter, and therefore, airborne spores can be readily inhaled by susceptible individuals (2). The disease predominantly affects immunocompromised patients, including those with uncontrolled diabetes mellitus, hematologic malignancies, or those receiving immunosuppressive therapy following organ transplantation (3, 4). Mucormycosis carries a strikingly high mortality rate, ranging from 50% to 100%, and has become the third most common invasive fungal infection in major U.S. transplant centers, following aspergillosis and candidiasis (1).

Accurate and timely diagnosis of mucormycosis remains a major clinical challenge. Conventional diagnostic methods, such as fungal culture and histopathology, often lack sensitivity and typically yield definitive results only in advanced stages of disease. This delay in diagnosis significantly limits the timely initiation of antifungal therapy, contributing to the poor outcomes associated with the disease (5). The recent surge of mucormycosis cases during the coronavirus disease 2019 (COVID-19) pandemic—particularly in India (6–10)—has further underscored the urgent need for rapid, sensitive, and specific diagnostic tools. Because of the high mortality rates associated with mucormycosis and currently inadequate diagnostic tools, the World Health Organization listed Mucorales fungi as a high-priority group in their first published fungal priority pathogen list (11–13).

Molecular diagnostic methods have emerged as promising alternatives to traditional techniques. Among these, loop-mediated isothermal amplification (LAMP) has gained considerable attention due to its simplicity, speed, and high sensitivity (14). LAMP amplifies DNA under constant temperature conditions (typically 60°C–65°C) using four to six specially designed primers that target six distinct regions of the DNA sequence (15). The reaction is driven by a strand-displacing DNA polymerase, enabling continuous and exponential amplification without the need for thermal cycling (14). As the reaction progresses, loop structures formed by displaced DNA strands facilitate further rounds of amplification, enabling rapid accumulation of target DNA (14). Detection of amplification can be achieved through fluorescence or colorimetric changes, offering both quantitative and visual readouts (16, 17).

**Fig 1:**
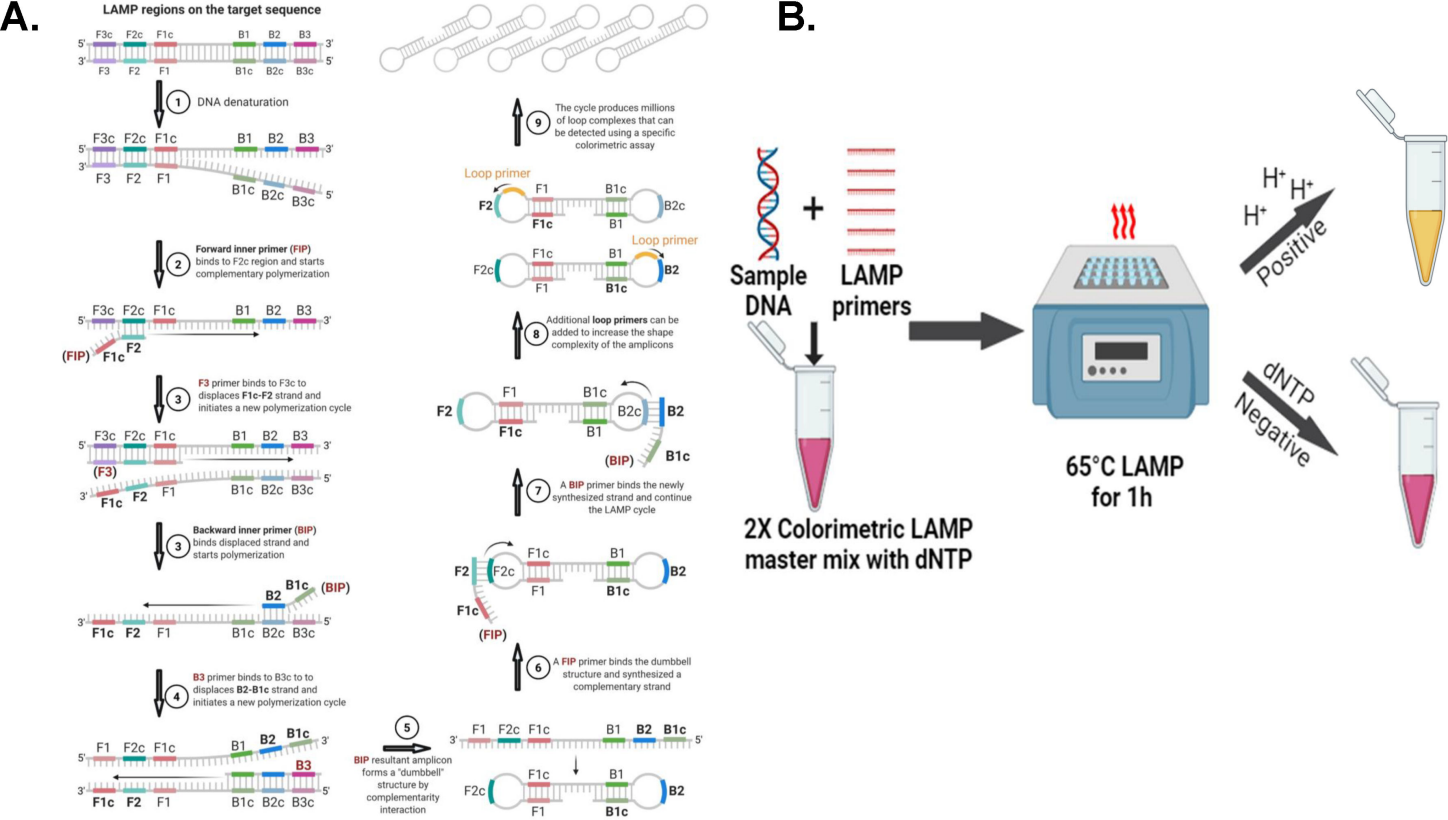
The schematic of the colorimetric LAMP assay for Mucorales DNA detection. (**A**) Mechanism of the traditional LAMP reaction. The reaction includes 4 to 6 primers: outer primers (F3 and B3), inner primers (FIP and BIP), and loop primers (LF and LB). Initially, FIP and F3 bind to the target sequence and initiate DNA strand synthesis, facilitated by Bst polymerase. Subsequently, BIP and B3 bind to their complementary sites and synthesize DNA on the opposite strand. The resulting amplicons contain complementary regions that self-hybridize, forming dumbbell-shaped structures that serve as templates for exponential amplification. (**B**) Workflow of the developed colorimetric LAMP assay incorporating phenol red as a pH-sensitive indicator, enabling rapid visual detection of Mucorales DNA based on color change.

In this study, we developed a colorimetric LAMP assay incorporating phenol red as a pH-sensitive indicator for the visual detection of Mucorales DNA (Fig. 1). The LAMP primer design was based on the Mucorales ribosomal DNA (rDNA) sequences, leveraging their high sequence conservation within the fungal group and lack of homology with mammalian (human and mouse) genomes or other types of fungi. This strategic targeting enhances assay specificity and minimizes cross-reactivity with host DNA. We evaluated the performance of the LAMP assay in both murine bronchoalveolar lavage (BAL) fluid samples and clinical BAL specimens from patients with mucormycosis and aspergillosis according to European Organization for Research and Treatment of Cancer/Mycoses Study Group Education and Research Consortium (EORTC/MSGERC) criteria (18).

## RESULTS

### Designing universal LAMP primers to target Mucorales fungi

To develop a broadly reactive LAMP assay for the detection of Mucorales fungi, we conducted a comparative analysis of the rDNA gene sequences from representative species, including *Rhizopus delemar*, *Mucor circinelloides*, *Lichtheimia corymbifera*, *Cunninghamella bertholletiae*, and *Rhizomucor*. Conserved regions were identified for primer design, while regions showing homology to the *Aspergillus fumigatus* (Af293) genome were excluded to ensure diagnostic specificity. Five initial LAMP primer sets targeting conserved 18S regions of *R. delemar* were designed with the online software (https://lamp.neb.com/#!/). All primers are proprietarily owned by Vitalex Bioscience, a company developing a LAMP-based diagnostic assay for mucormycosis. These sets were subsequently aligned against conserved rDNA sequences from other Mucorales species to assess coverage and sequence compatibility. LAMP162 exhibited the highest degree of conservation, with only a single nucleotide mismatch in the forward inner primer (FIP) across all target species. Thus, degenerate bases were incorporated into the FIP primer to maximize sensitivity while maintaining specificity.

**Fig 2:**
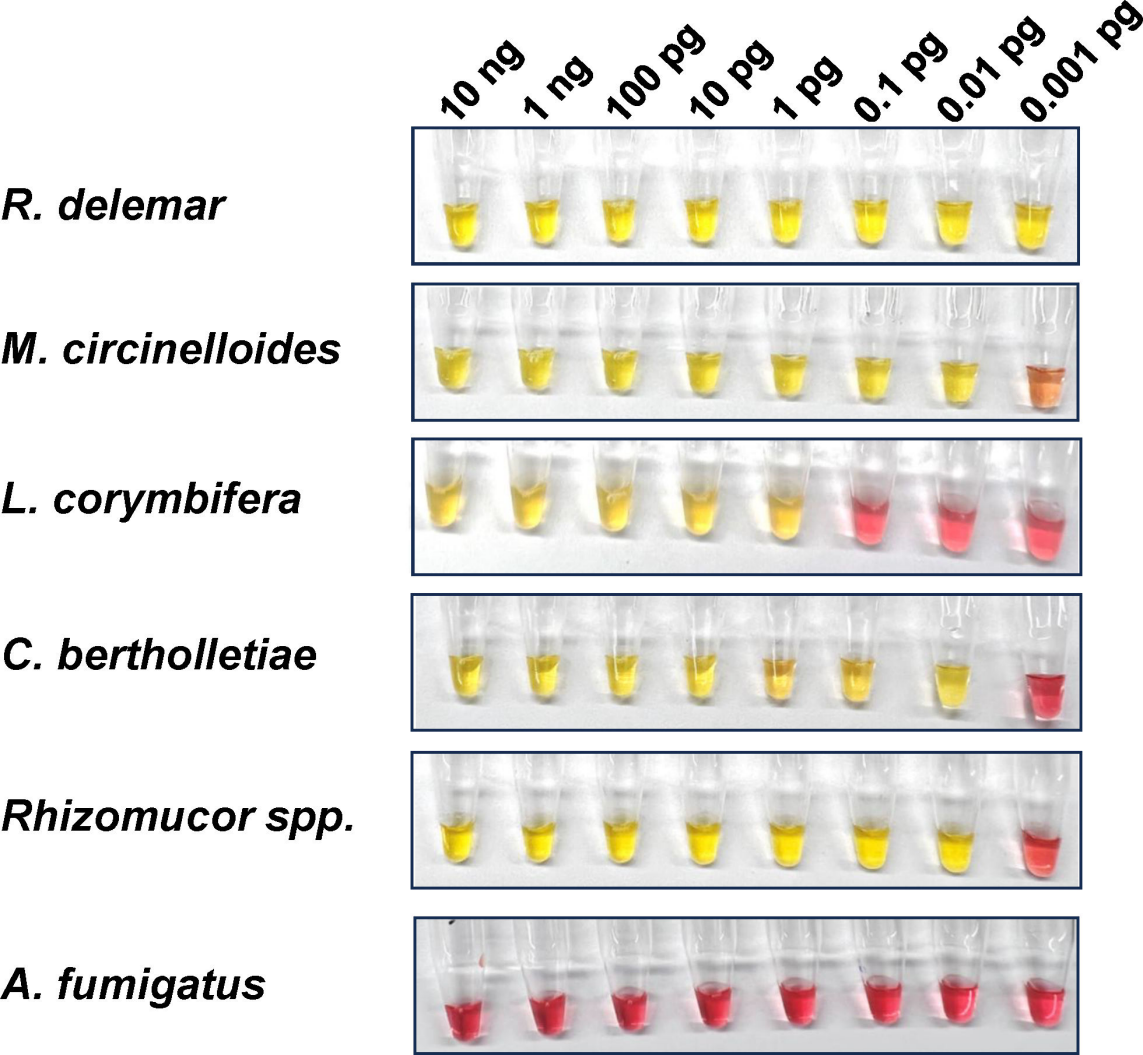
Sensitivity and specificity of the colorimetric LAMP assay for Mucorales DNA detection. The assay was evaluated using spiked genomic DNA from various Mucorales species and *A. fumigatus* as a specificity control. The LAMP assay successfully detected as little as 0.001 pg of *R. delemar* DNA, 0.01 pg of *M. circinelloides*, *C. bertholletiae*, and *Rhizomucor* spp. DNA and 1 pg of *L. corymbifera* DNA. No amplification was observed for *A. fumigatus* DNA across a range of 0.001 pg to 10 ng, confirming the assay’s specificity.

The analytical sensitivity and specificity of the LAMP assay were evaluated using purified genomic DNA from *R. delemar*, *M. circinelloides*, *L. corymbifera*, *C. bertholletiae*, *Rhizomucor* spp., and *A. fumigatus* Af293. Reactions were performed using the New England Biolabs (NEB) colorimetric LAMP master mix and incubated for 60 min with serial dilutions of fungal DNA. The assay demonstrated robust sensitivity, detecting <0.001 pg of *R. delemar* DNA, 0.01 pg of *M. circinelloides*, 1 pg of *L. corymbifera*, 0.01 pg of *C. bertholletiae*, and 0.01 pg of *Rhizomucor*. Based on an estimated genome mass of approximately 0.04 pg (19) per Mucorales genome (average size of *R. delemar, M. circinelloides,* or *Lichtheimia* of ~40 Mb NCBI data base), these detection limits correspond to ~0.025, 0.25, 25, 0.25, and 0.25 CFU equivalents, respectively. Because the assay targets the multicopy rDNA region, the actual number of detectable rDNA target copies is substantially higher; for example, assuming 100 rDNA copies per genome, the assay can detect roughly 2–3 rDNA target copies for *R. delemar* and ~25 copies for *M. circinelloides*, *C. bertholletiae*, and *Rhizomucor*. No amplification was observed with *A. fumigatus* DNA, confirming the assay’s high specificity for members of the order Mucorales (Fig. 2).

### Detection of Mucorales infection from mouse biological samples

**Fig 3:**
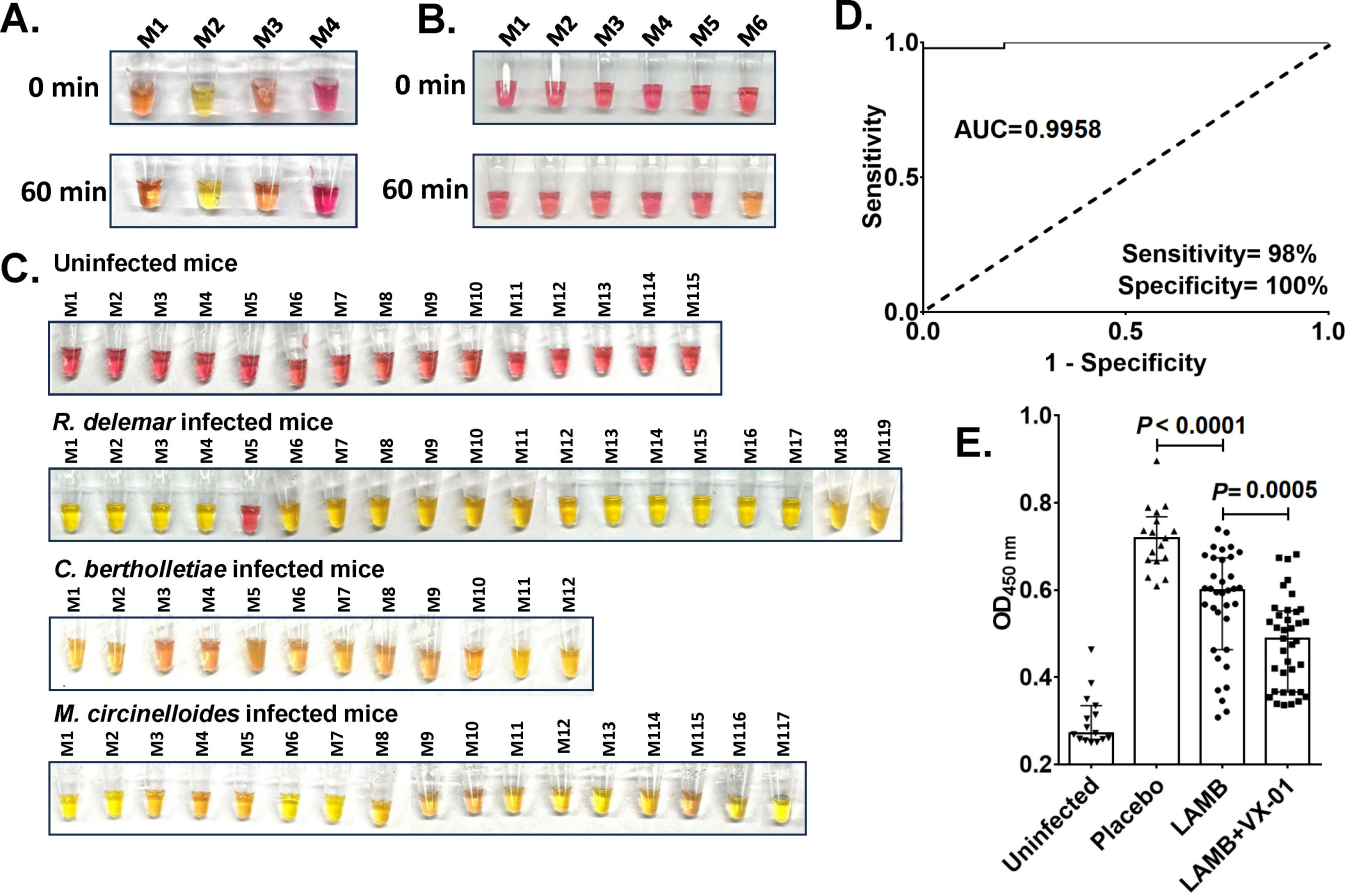
Evaluation of the colorimetric LAMP assay using biological samples from Mucorales-infected mice. (**A**) Urine samples from infected mice yielded inconsistent results, indicating that urine is not a suitable specimen for LAMP-based detection. (**B**) Serum samples from infected mice also failed to provide reliable LAMP signals. (**C**) The assay successfully detected fungal DNA in BAL samples collected from infected mice. Positive LAMP results (yellow color) were obtained for 18 of 19 *R. delemar*-infected, 12 of 12 *C. bertholletiae*-infected, and 17 of 17 *M. circinelloides*-infected animals. BAL samples were collected at the onset of illness, typically 1–4 days post-infection. All BAL samples from uninfected mice showed negative DNA amplification (pink color). (**D**) Receiver operating characteristic (ROC) curve analysis of BAL testing demonstrated excellent diagnostic performance, with an area under the curve (AUC) of 0.996, sensitivity of 98%, and specificity of 100%. (**E**) Intensity of the LAMP assay yellow color development (measured as optical density at 450 nm [OD_450 nm_]) of mouse BAL samples infected with *R. delemar* and treated with liposomal amphotericin B (LAMB), or a combination of LAMB+VX-01 MAb correlated with historical protective effect of LAMB compared to placebo mice (lower OD_450 nm_ readings for LAMB-treated vs placebo), and the enhanced effect of combination therapy of LAMB+VX-01 (lowest OD_450 nm_ readings).

Following the promising performance of the LAMP assay with spiked genomic DNA, we next evaluated its diagnostic utility using biological specimens derived from a neutropenic murine model of mucormycosis. We initially assessed the feasibility of using urine as a non-invasive sample type. However, due to variability in urine pH, false-positive signals were observed immediately after reaction setup in samples from uninfected mice (Fig. 3A). We also tested the ability of the LAMP assay to amplify DNA from sera collected from mice at varying time points following intratracheal (IT) infection. However, the assay did not amplify any DNA (Fig. 3B), potentially because of the reported inhibitory effects of blood and serum components on DNA amplification (20). These limitations led us to shift our focus to BAL fluid, a more reliable and clinically relevant sample type for pulmonary mold infection (3, 21). BAL fluid directly samples the initial site of infection, increasing the likelihood of detecting fungal elements and improving assay accuracy (22). A total of 48 BAL samples were obtained from Mucorales-infected mice: 19 infected with *R. delemar*, 12 with *C. bertholletiae*, and 17 with *M. circinelloides*. Additionally, 15 BAL samples from uninfected mice were included as negative controls. Of the infected samples, 47 tested positive in the LAMP assay, with only one false negative observed in a mouse from the *R. delemar*-infected group. All BAL samples from uninfected mice tested negative, highlighting the high specificity of the LAMP assay (Fig. 3C).

To quantitatively assess diagnostic performance, we conducted ROC curve analysis using OD_450 nm_ values measured from reactions transferred to a 384-well plate. Positive reactions yielded high OD_450 nm_ values, while negative samples exhibited low readings. The assay demonstrated a sensitivity of 98% and a specificity of 100%, with an AUC of 0.9958, indicating excellent discriminatory power for mucormycosis diagnosis using BAL fluid (Fig. 3D).

Having established the diagnostic performance of the LAMP assay in detecting Mucorales DNA from infected murine BAL samples, we next sought to evaluate its ability to monitor antifungal treatment efficacy. Since antifungal therapy is expected to reduce the fungal burden, this approach enables assessment of whether the LAMP assay signal correlates with infection progression and therapeutic response, key features for its potential use as a treatment-monitoring tool in clinical settings. In our laboratory, we developed a humanized monoclonal antibody, VX-01, targeting the Mucorales-specific surface protein CotH3. Previous studies demonstrated that combining VX-01 with LAMB, the standard-of-care antifungal therapy for mucormycosis, significantly improved survival in a murine model versus placebo or monotherapy (23). To investigate whether the LAMP assay can detect reductions in fungal DNA following treatment, we performed a separate experiment in which neutropenic mice were infected with *R. delemar* and treated 24 h post-infection. Mice received either VX-01 (30 µg, single dose, i.v.), LAMB (10 mg/kg, i.v. for 4 days), or a combination of both. BAL samples were collected from each group at multiple time points for subsequent LAMP analysis. The LAMP assay revealed a significant reduction in *R. delemar* DNA levels in BAL fluid from mice treated with the combination of LAMB+VX-01, compared to those treated with LAMB alone or placebo (Fig. 3E). These findings confirm the enhanced antifungal efficacy of combination therapy and demonstrate the potential use of the assay not only for early diagnosis of mucormycosis but also as a quantitative tool for monitoring treatment response.

### Validation of the LAMP assay using human BAL samples

Following the successful application of the LAMP assay with the murine model, we next evaluated its performance in human clinical samples. BAL specimens were obtained from 24 patients with proven mucormycosis and 17 patients diagnosed with either proven aspergillosis or confirmed to be negative for mucormycosis. Detailed clinical characteristics of these patients are provided in Table 1.

**TABLE 1:** Clinical characteristics of patients with proven mucormycosis or aspergillosis from whom BAL samples were collected for LAMP assay evaluation^*a*^

LAMP ID	EAD	Age	Sex	BAL fungal culture	Result MucorGenius	Sample type PCR	Proven/not Mucormycosis	LAMP result
LAMP1	87584652	63	Female	No mold	Positive	BAL	Proven	Positive
LAMP2	70673967	68	Male	No mold	Positive	BAL	Proven	Positive
LAMP3	61551351	74	Male	*No mold*	Positive	BAL	Proven	Positive
LAMP4	77655637	57	Female	*Rhizopus species*	Not performed	N/A	Proven	Positive
LAMP5	71357354	57	Male	*Rhizopus microsporus*	Not performed	N/A	Proven	Positive
LAMP6	61900947	20	Female	*Rhizopus microsporus*	Not performed	N/A	Proven	Negative
LAMP7	70782610	45	Male	*Rhizopus arrhizus*	Not performed	N/A	Proven	Positive
LAMP8	60924190	58	Male	*Rhizopus microsporus*	Not performed	N/A	Proven	Positive
LAMP9	82744244	64	Female	*Lichtheimia species*	Not performed	N/A	Proven	Positive
LAMP10	61067814	9	Male	*Rhizomucor pusillus*	Not performed	N/A	Proven	Positive
LAMP11	82153982	66	Male	*Rhizomucor pusillus*	Not performed	N/A	Proven	Positive
LAMP12	71865059	69	Male	*Lichtheimia species*	Not performed	N/A	Proven	Negative
LAMP13	75076257	61	Male	*Rhizomucor pusillus*	Not performed	N/A	Proven	Positive
LAMP14	87415881	77	Female	*Lichtheimia ramosa*	Not performed	N/A	Proven	Positive
LAMP15	60748607	64	Male	*Mucor circinelloides*	Negative	Tissue	Proven	Negative
LAMP16	72089428	64	Female	*Rhizopus species*	Positive	BAL	Proven	Positive
LAMP17	86714185	66	Female	*Rhizopus arrhizus*	Negative	Tissue	Proven	Positive
LAMP18	77465268	33	Male	Calcofluor positive, possible Mucorales	Positive	BAL	Proven	Positive
LAMP19	62938962	81	Male	No mold	Positive	BAL	Proven	Negative
LAMP20	77443950	76	Female	No mold	Positive	BAL, serum	Proven	Positive
LAMP21	2744	48	Female	*Lichtheimia corymbifera*	Positive	BAL	Proven	Negative
LAMP22	2648	46	Male	No mold	Positive	BAL	Proven	Positive
LAMP23	Biz	53	Male	*Rhizopus microsporus*	Not performed	BAL	Proven	Positive
LAMP24	R14 936	65	Female	*Aspergillus fumigatus*	Positive	BAL	Proven	Positive
LAMP25	H042	52	Female	No mold	Not performed	N/A	Not	Negative
LAMP26	H044	26	Male	No mold	Not performed	N/A	Not	Positive
LAMP27	IFI066	69	Male	No mold	Not performed	N/A	Not	Negative
LAMP28	IFI078	20	Male	No mold	Not performed	N/A	Not	Negative
LAMP29	IFI079	36	Female	No mold	Not performed	N/A	Not	Negative
LAMP30	IFI080	44	Female	No mold	Not performed	N/A	Not	Negative
LAMP31	IFI081	30	Female	No mold	Not performed	N/A	Not	Negative
LAMP32	IFI086	48	Female	No mold	Not performed	N/A	Not	Negative
LAMP33	IFI087	44	Male	No mold	Not performed	N/A	Not	Negative
LAMP34	IFI089	39	Male	No mold	Not performed	N/A	Not	Negative
LAMP35	IFI098	79	Male	No mold	Not performed	N/A	Not	Negative
LAMP36	IFI107	38	Male	No mold	Not performed	N/A	Not	Negative
LAMP37	IFI113	69	Female	No mold	Not performed	N/A	Not	Negative
LAMP38	IFI116	48	Female	No mold	Not performed	N/A	Not	Negative
LAMP39	IFI124	30	Female	No mold	Not performed	N/A	Not	Negative
LAMP40	R17 1036	75	Male	*Aspergillus fumigatus*	Negative	BAL	Not	Negative
LAMP41	R18 1047	63	Female	*Aspergillus fumigatus*	Negative	BAL	Not	Negative

^*a*^ This table summarizes detailed clinical information for patients diagnosed with mucormycosis or aspergillosis, including diagnostic classification, underlying conditions, site of infection, and fungal species (if available). These patient BAL samples were used to assess the diagnostic performance of the colorimetric LAMP assay described in this study. N/A, not applicable for PCR test. Grey shading highlights negative LAMP test.

**Fig 4:**
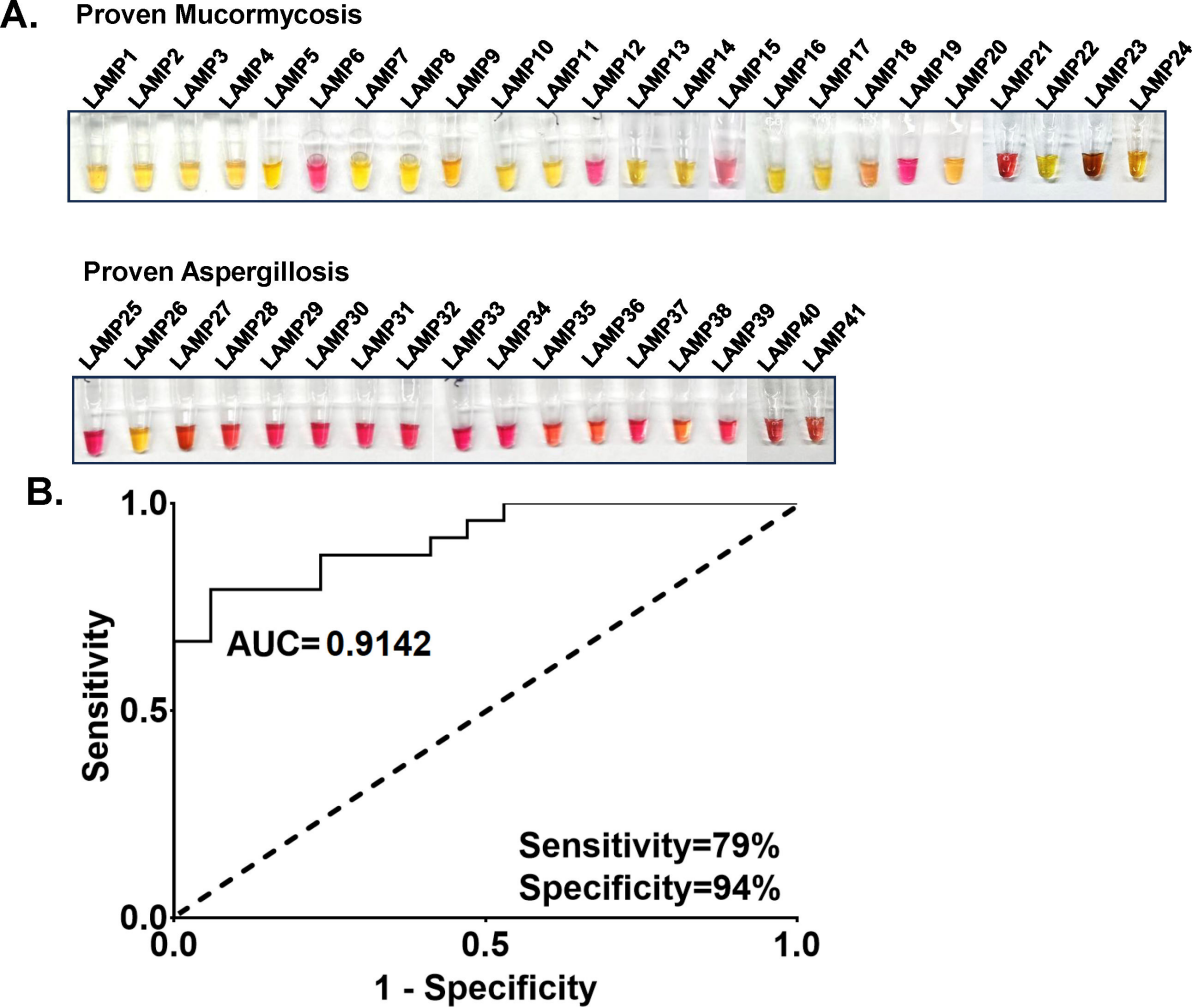
Application of the colorimetric LAMP assay for detecting Mucorales DNA in human clinical BAL samples. (**A**) Among 24 BAL samples from patients with confirmed mucormycosis, 19 tested positive using the LAMP assay. In contrast, 16 out of 17 BAL samples from patients with aspergillosis remained negative, demonstrating the assay’s clinical specificity. (**B**) ROC curve analysis for the clinical samples yielded an AUC of 0.9142, indicating robust diagnostic performance. The sensitivity and specificity of the LAMP assay in this cohort were 79% and 94%, respectively.

When applied to the human BAL samples, the LAMP assay yielded positive results in 19 of 24 mucormycosis cases with one sample (LAMP23) displaying brown color rather than yellow because of the presence of infected tissue remnants (Fig. 4A). Among the 17 mucormycosis-negative samples, only one tested false positive, demonstrating a high level of specificity (Fig. 4A). ROC curve analysis showed the assay achieved a sensitivity of 79% and a specificity of 94%, with an AUC of 0.9142 (Fig. 4B). These findings support the diagnostic potential of the LAMP assay for use in clinical settings, offering a rapid and reliable molecular tool for the detection of mucormycosis in human BAL specimens.

## DISCUSSION

With the lack of currently approved serological tests, the current diagnosis of mucormycosis relies heavily on culturing the organism from otherwise sterile body fluid and/or tissue histology (24). Both methods have shortcomings and have little influence on how the disease is managed. For example, culturing methods can lead to false-positive results because Mucorales fungi are ubiquitous in nature and can easily contaminate sterile samples during laboratory processing. Even worse, culturing of biopsies can lead to false negative results due to the frequently used procedure of tissue biopsy homogenization, which damages hyphae, leading to a lack of fungal growth on cultured media (24). In this scenario, an infected patient can go untreated for a disease with high mortality rates. Similarly, relying on histology alone is often associated with delayed diagnosis because the infection has already spread and caused extensive tissue necrosis. Tissue necrosis is associated with poor antifungal drug delivery to the site of infection (9, 23). Clearly, more effective diagnostic tools for mucormycosis are needed.

Recently, molecular diagnostic assays have emerged as promising, reliable tools for the diagnosis of mucormycosis. Specifically, real-time PCR-based approaches detecting Mucorales DNA in fresh or formalin-fixed paraffin-embedded tissues, as well as in BAL fluid or blood, have been described largely using either BAL fluid or blood samples targeting rDNA (25–27). The sensitivity and specificity of these assays are generally high (90% of sensitivity and >95% specificity among proven mucormycosis according to the EORTC/MSG criteria [18]). Additionally, these assays were shown to discriminate among the genera causing mucormycosis with the ability to predict infection days before diagnosis using conventional methods (25, 27).

In the MODIMUCOR prospective trial, it was shown that a Mucorales-specific qPCR showed 85.2% sensitivity and 89.8% specificity in detecting Mucorales DNA in serum samples as early as a median of 4 days (interquartile range [IQR], 0–9) before sampling of the first mycological positive specimen and a median of one day (IQR, −2 to 6) before the first imaging was performed (28). Interestingly, some of these qPCR assays were shown in retrospective analysis to have prognostic potential, showing that patients with negative DNA amplification following treatment had better survival outcomes compared to patients who remained positive for DNA amplification (28). A more recent study prospectively conducted on 744 patients who had 35 proven aspergillosis cases, 16 mucormycosis cases, and four cases with co-infections of *Aspergillus*/Mucorales showed that the MycoGenie qPCR kit (Adamtech, Pessac, France) was able to effectively differentiate between the two infections with 80% sensitivity for detecting Mucorales DNA in serum samples (29). Finally, we also reported on the use of the conventional PCR method to amplify CotH, which is a gene family specific to Mucorales and encodes proteins that enable these fungi to invade host tissues. The assay amplified CotH DNA from biological samples from mice with mucormycosis, showing a higher sensitivity of 90% and specificity of 100% in urine samples (30). The assay was validated by amplifying DNA from urine samples collected from four patients with proven mucormycosis with 100% sensitivity (30). Collectively, these studies show that PCR-based diagnostic assays can be used reliably for early detection of mucormycosis and can be used to predict therapy outcome. However, drawbacks of relying on PCR-based assays remain and include the time consumption necessitated by DNA extraction methods, the need for skilled laboratory personnel to perform these extractions and operating machines, and the relatively long turnaround times in real-world settings, as well as the cost associated with these molecular assays.

The non-PCR-based LAMP assay offers a simple and quick diagnostic alternative while maintaining the high degree of sensitivity and specificity shown by conventional or real-time PCR assays. One of the major advantages of the LAMP assay is its colorimetric readout, which produces a visible color change upon amplification. Equally important, the assay eliminates the need to use complicated machines and trained personnel and requires minimum handling of the sample because there is no need for a DNA extraction step, thereby reducing the chance of contaminating the sample. These features allow for easy interpretation of results, making the assay particularly suitable for deployment in low-resource environments or point-of-care settings.

In this study, our results demonstrate that the LAMP assay using degenerate primers designed to target conserved regions of rDNA across multiple Mucorales species offers robust performance with high sensitivity and specificity. The assay reliably detected minute quantities of fungal DNA in both controlled experimental settings and clinical specimens. Importantly, the assay did not detect DNA from any samples spiked with *A. fumigatus* or from samples taken from patients diagnosed with invasive pulmonary aspergillosis (Table 1). Given the overlapping clinical presentations of mucormycosis and aspergillosis (3, 9), and also given their frequent co-occurrence (31), distinguishing between these pathogens is critical for early and appropriate antifungal therapy. In samples spiked with *R. delemar* DNA, the assay can detect DNA at concentrations of <0.4 femtogram/10 µL, a sensitivity that is ~10-fold higher than what has been reported using qPCR (DNA detectable concentrations of 3.7–15 femtogram/10 µL) (25). This sensitivity was reduced by 10-fold to 4.0 femtogram/10 µL when the samples were spiked with *M. circinelloides, C. bertholletiae,* or *Rhizomucor*. The assay was least sensitive with samples spiked with *L. crymbifera* DNA (sensitivity of 400 femtograms/10 µL). Interestingly, it appears that at least in one study that used qPCR targeting rDNA was also the least sensitive in identifying mucormycosis using serum samples infected with *L. corymbifera* (25).

In a murine model infected with *R. delemar*, the assay achieved a sensitivity of 98% and specificity of 100% using BAL samples, effectively distinguishing it from other fungal infections, such as invasive pulmonary aspergillosis. Also, like the PCR-based diagnostic assay which showed potential for being used to predict treatment outcome (28), we show that the use of a synergistic treatment regimen of VX-01+LAMB against *R. delemar*-induced murine mucormycosis enhanced overall survival (23) and reduced the amount of amplified DNA (Fig. 3E). In human patient BAL samples, the assay also demonstrated strong diagnostic accuracy, with an AUC of 0.9142 and 79% overall sensitivity. This sensitivity was similar to an 83% sensitivity reported using LAMP primers to amplify CotH in serum samples of 30 patients diagnosed with mucormycosis (32).

A potential limitation of this study is the use of human BAL specimens from archived frozen samples. Although fungal DNA is generally stable at −80°C, prolonged storage or repeated freeze-thaw cycles may lead to partial degradation of nucleic acids, which could contribute to occasional false-negative results. Nevertheless, the high diagnostic sensitivity and specificity observed in this study suggest that any effect of storage on assay performance was minimal. Future prospective studies using freshly collected BAL samples will further validate the assay’s clinical utility under routine diagnostic conditions.

Another limitation of the study is the fact that the current assay using degenerate primers does not distinguish among the various causative genera of mucormycosis. Therefore, this assay is considered a pan-Mucorales assay detecting members of the order Mucorales without identification of the causative Mucorales agent. While the lack of ability to differentiate between Mucorales members limits species-level epidemiological analyses, the assay provides a reliable and rapid diagnostic tool for early detection and clinical management of mucormycosis. It is imperative to emphasize that this assay shortcoming has no immediate effect on how the disease is treated. Also, the assay in its current nature is semi-quantitative rather than quantitative. Finally, the assay in its current form does not work very well for serum samples. Consequently, its use for non-pulmonary manifestations of the disease might be limited. Nonetheless, our results highlight the high sensitivity and specificity of this assay, demonstrating its potential as a robust molecular diagnostic platform for the early detection of mucormycosis. Thus, the LAMP assay presented in this study represents a promising diagnostic tool for mucormycosis. Its simplicity, rapid turnaround time, and high diagnostic performance make it a valuable addition to current diagnostic strategies. By enabling early detection, especially in high-risk populations and in settings with limited laboratory infrastructure, the LAMP assay has the potential to improve clinical outcomes and reduce the burden of this devastating infection.

## MATERIALS AND METHODS

### Fungal strains

Five clinically isolated Mucorales strains were used to test the sensitivity of the LAMP assay. These included *R. delemar* 99-880 and *M. circinelloides* (obtained from the Fungus Testing Laboratory at the University of Texas Health Sciences Center at San Antonio), *L. corymbifera* 008-0490 (obtained from a patient that was enrolled in the DEFEAT Mucor clinical trial) (33), *C. bertholletiae* 182 (a gift from Dr. Thomas Walsh), and *Rhizomucor* (obtained from a patient at the Harbor-UCLA Medical Center). For validation of the specificity of the assay, *Aspergillus fumigatus* (Af293) was used as the negative control. *R. delemar, C. bertholletiae, L. corymbifera,* and *Rhizomucor* were grown on potato dextrose agar, and Af293 and *M. circinelloides* were grown on yeast peptone dextrose agar. The genomic DNA from all the strains was extracted from hyphae grown in potato dextrose broth overnight as previously described (30) and quantified using Nanodrop.

### LAMP primer design

The rDNA of the Mucorales fungi strains was blasted through Clone Manager to identify the most conserved region. The Mucorales rDNA was also blasted against the *Aspergillus*, mouse, and human genomes to ensure the lack of similarities to these targets. Proprietary LAMP primers were designed to target the conserved regions of 18S rDNA with the NEB LAMP primer design tool: https://lamp.neb.com/#!/. The sensitivity and specificity of the assay were evaluated using Mucorales and *Aspergillus* spiking DNA samples of known concentrations, ranging from femtograms to picograms.

### LAMP assay setting

The LAMP assay was performed using 12.5 µL of 2× colorimetric LAMP Master Mix containing phenol red (NC1380779, Fisher Scientific) in a 0.5 mL PCR tube. Water (9 µL) was added to the tube, followed by 2.5 µL LAMP primer mix, followed by 1 µL of either spiked DNA samples, mouse urine, serum, or BAL fluid, or patient BAL samples, and the 2× colorimetric LAMP Master Mix. The isothermal amplification was carried out at 65°C for 60 min, resulting in a total turnaround time of approximately 1 h from sample addition to visual readout. A clear visual detection of amplification based on the production of protons and a subsequent drop in pH that occurs from the extensive DNA polymerase activity in a LAMP reaction, the distinctive color change of phenol red from pink to yellow indicates a positive reaction, simplifying result interpretation without the need for specialized equipment. For BAL samples, only 1 µL of the sample was used per reaction, which was sufficient for sensitive detection of Mucorales DNA.

### Mouse model

Male ICR mice weighing 20–23 g were obtained from Envigo for all animal experiments. Neutropenia was induced by intraperitoneal (IP) injection of cyclophosphamide (200 mg/kg) and subcutaneous injection of cortisone acetate (500 mg/kg) on days −2 and +3, relative to infection. IT infection with different Mucorales was conducted as detailed before (34). Urine, serum, and BAL samples were collected at various time points post-infection. The LAMP assay was applied to these BAL samples to assess the sensitivity of the assay. Biological samples obtained from uninfected control mice were included to validate the specificity of the assay. To determine the possibility of the assay in predicting treatment outcome, we collected biological samples from mice IT infected with *R. delemar* as above and treated with proven effective therapy of a single dose of a humanized anti-CotH3 antibody (VX-01) administered IP at 30 µg alone or combined with intravenous administration of liposomal amphotericin B (LAMB, Gilead Sciences) at 10 mg/kg/day given for 4 days (23, 34). Treatment started 24 h post-infection. Biological samples were collected from mice at different time points post-infection and were used in the LAMP assay as above.

### Human samples

To further validate our assay, human biological samples were included in the analysis and obtained from Department of Laboratory Medicine, National Reference Center for Mycosis, University Hospitals Leuven (Leuven, Belgium), Division of Infectious Diseases, Department of Medicine, Medical University of Graz (Graz, Austria), and Division of Infectious Diseases, University of California San Diego (San Diego, USA). All samples were collected after obtaining approval at each of the institutions’ respective institutional review boards (35, 36) and were tested in the LAMP assay at The Lundquist Institute under IRB protocol 33179-01 without using identifiers.

### Statistical analysis

The sensitivity and specificity of the LAMP assay were evaluated using the ROC curve analysis, which visualizes the sensitivity and specificity characteristics of a particular assay (37, 38). The optical density 450 nm (OD_450 nm_) of each LAMP assay after the reaction was obtained; typically, the negative samples had lower OD readings, and the positive samples that turned yellow had high OD readings. The y-axis of the ROC graph represents sensitivity or the true positive rate. The x-axis represents the component complement of specificity (100%—specificity). An AUC is a commonly used measure, where an AUC of 1.0 represents a perfect curve fit, while an AUC of 0.5 represents random classification. Calculations of the ROC curve and AUC were performed with Prism GraphPad. For validating the efficacy of the antifungal treatment between the LAMB, VX-01, or the combination of both, differences in OD_450 nm_ reading of the LAMP reaction from each treatment group were compared by the non-parametric Mann-Whitney (two-tailed) test for multiple comparisons. *P* < 0.05 was considered significant.

## Data Availability

All data are available in the main text of this manuscript. Primer sequences are proprietarily owned by Vitalex Bioscience, which is aiming to make the assay available for clinical use through commercialization. The sequences are available upon request from the corresponding author and after signing a Material Transfer Agreement (MTA) stating that the primers will be used strictly for research purposes and not for profit or commercial development.
